# The Role of NLRP3 and IL-1β in Refractory Epilepsy Brain Injury

**DOI:** 10.3389/fneur.2019.01418

**Published:** 2020-02-07

**Authors:** Chunfeng Wu, Gang Zhang, Lei Chen, Samuel Kim, Jie Yu, Guo Hu, Jing Chen, Yanjun Huang, Guo Zheng, Songming Huang

**Affiliations:** ^1^Department of Neurology, Children's Hospital of Nanjing Medical University, Nanjing, China; ^2^Department of Physiology, Nanjing Medical University, Nanjing, China; ^3^Department of Anesthesiology, Emory University School of Medicine, Atlanta, GA, United States; ^4^Department of Nephrology, Children's Hospital of Nanjing Medical University, Nanjing, China

**Keywords:** refractory temporal lobe epilepsy, inflammatory factor, NLRP3, IL-1β, brain injury

## Abstract

**Objective:** The objective of this study was to investigate the roles and mechanisms of inflammatory mediators NLRP3 and IL-1β in refractory temporal epilepsy brain injury.

**Method:** First, the brain tissue and the peripheral blood of children undergoing intractable temporal lobe epilepsy surgery were analyzed as research objects. The expression levels of NLRP3 in brain tissue and IL-1β in blood were measured. A model of temporal lobe epilepsy was established using wild-type and NLRP3 knockout 129 mice. Pilocarpine was injected intraperitoneally into the experimental group, and isovolumetric saline was injected intraperitoneally into the control group (*n* = 8 in each group). The expression of IL-1β in the peripheral blood, cerebral cortex, and hippocampus of mice was measured by ELISA at 3 h, 24 h, 3 days, and 7 days after modeling. Fluoro-Jade B (FJB) and TUNEL methods were used to determine necrosis and apoptosis in hippocampal neurons, respectively, and the expression of NLRP3 in the cortex was measured by immunofluorescence methods.

**Result:** (1) The IL-1β levels in the peripheral blood of children with intractable temporal lobe epilepsy were higher than those in the control group (*t* = 2.813, *P* = 0.01). There was also a positive correlation between IL-1β expression levels and the onset time of a single convulsion in patients with refractory epilepsy (*r* = 0.9735, *P* < 0.05). The expression level of NLRP3 in the cerebral cortex of patients with refractory temporal lobe epilepsy was higher than that in the control group. (2) The expression level of NLRP3 in the hippocampus of wild-type mice increased 3 days after modeling and decreased slightly at 7 days but remained higher than that of the control group. IL-1β levels in peripheral blood were significantly higher than those in the control group at 3 days (*t* = 8.259, *P* < 0.0001). The IL-1β levels in the peripheral blood of NLRP3 knockout mice were lower than those in the wild-type group at 3 days (*t* = 3.481, *P* = 0.004). At day 7, the neuronal necrosis and apoptosis levels in the CA3 region of the hippocampus decreased.

**Conclusion:** NLRP3 may be involved in the development of refractory temporal lobe epilepsy. Inhibiting NLRP3 may alleviate local brain injury by downregulating the IL-1β expression. The IL-1β levels in the peripheral blood of patients with refractory temporal lobe epilepsy may reflect the severity of convulsions.

## Introduction

Refractory epilepsy (RE) refers to epilepsy in which patients still have seizures after being treated with two kinds of antiepileptic drugs that are correctly selected and can be tolerated. RE can easily induce permanent brain damage, especially neuronal death within the hippocampus (1). RE also leads to metabolic disorders in human tissue cells, depleted glucose and oxygen, and ion membrane transport disorder, further exacerbating damage to neurons (2–4). Repeated seizures promote inflammatory reactions, which induce free radical production, neuronal metabolism overload, and increased excitatory glutamate. Increased glutamate levels can potentially injure neurons and further aggravate seizures (5). Some investigators have observed increased levels of peripheral neutrophils, plasma albumin, and the inflammatory factor IL-1β in patients with recurrent seizures (6). Additionally, the anti-inflammatory drug dexmedetomidine can downregulate IL-1β expression and that of other inflammatory mediators to reduce seizures and brain damage in epileptic mice. FK506 ameliorated the course of pilocarpine-induced epilepsy and the neuronal loss in the rat hippocampus after SE (7, 8). However, the regulatory mechanism of inflammatory mediators in RE patients is still unclear.

The NLRP3 inflammatory corpuscle is a protein complex in the body that promotes the release of inflammatory factors in response to infection or tissue injury. NLRP3 inflammatory corpuscles are known to be associated with many nervous system diseases, such as Alzheimer's disease (AD), neurodegenerative diseases, and depression (9–11). Under normal circumstances, inflammatory corpuscles exist in an inactive form in microglia and astrocytes (12). When cells are subjected to specific stimuli, such as hypoxia or complement-mediated injury, NLRP3 inflammatory corpuscles can be activated to promote the production of active IL-1β (13). The purpose of this study was to investigate the mechanism of NLRP3 action in the treatment of intractable temporal lobe epilepsy brain injury.

## Materials and Methods

### Cases and Specimen Collection

Data from seven children (three males and four females) with intractable temporal lobe epilepsy who were admitted to the epilepsy center of Nanjing Brain Hospital from April 2016 to December 2016 for surgical treatment were used. Epilepsy was diagnosed according to the ILAE epilepsy classification of 2015. All children had a history of more than half an hour of seizures and had not been cured with the administration of two or more antiepileptic drugs. All patients' peripheral serum was collected before their operations, and the peripheral blood of healthy examined children was used as the serum sample control. Epileptogenic zone brain tissues were collected, and normal brain tissues adjacent to this zone were collected as the control. This study was approved by the Ethics Committee of the Brain Hospital, and informed consent was obtained from the parents of the patients. The basic information and diagnoses of the seven children with intractable epilepsy are shown in Table 1.

**Table 1:** Clinical data of patients.

**Number**	**Gender**	**Age (years)**	**Disease course (years)**	**Duration of a single seizure (min)**	**Diagnosis**	**Histopathological results**
1	F	15	6	40	TE	G
2	M	12	3	30	TE	G, NL
3	F	16	4	60	TE	G, ND
4	M	14	8	45	TE	G, NL
5	F	16	6	50	TE	G, ND
6	M	12	4	30	FE	G, ND
7	M	13	5	35	TE	G

*TE, Temporal lobe epilepsy; FE, frontal lobe epilepsy; G, gliosis; NL, neuronal loss; ND, neuron degeneration*.

### Animal Grouping and Modeling

In this investigation, 129 wild-type (WT) and gene knockout (NLRP3^−/−^) male mice ~10 weeks old and weighing ~30 g were used (Jackson Company, USA). Mice were randomly divided into either the model group or the control group. The model group was given 1 mg/kg scopolamine intraperitoneally. After 15 min, 300 mg/kg pilocarpine was injected intraperitoneally (14). The control group received intraperitoneal injections of isovolumetric saline. Mice were sacrificed at 3 h, 24 h, 3 days, and 7 days after modeling, with eight mice in each group. Blood and brain tissue samples were also collected. Behavioral scores after drug administration were graded according to the classic Racine V epilepsy behavior standard. The scoring was based on the Racine scale, as described previously (15). Those that scored above grade IV were considered successfully modeled.

### Specimen Preparation and Material Selection

The convulsions of all mice were recorded before each observation time point. Mice were then anesthetized with 3.5% chloral hydrate. After death, blood was collected from the left heart cavity within 4 h and centrifuged at 4°C and 1,500 rpm for 10 min. The supernatant was used to detect inflammatory factors by ELISA. Additionally, mice were simultaneously divided into either a fresh ice group or a perfusion group. In the fresh ice group, animal brain tissue was taken from the ice tray placed in an EP tube, marked, and then stored in a −80°C refrigerator. In the perfusion group, mice were perfused with 4% paraformaldehyde, and brain tissue was stored in paraformaldehyde, dehydrated, and embedded in paraffin for immunohistochemical detection.

### Western Blot Analysis

The mouse brain tissue samples were added to the lysate ice and allowed to stand for 15 min and were then centrifuged at 15,000 rpm for 30 min at 4°C. The supernatant was transferred to a new EP tube. The protein concentration was determined using the BCA method; the quantitative protein was added into a 5 × SDS loading buffer in proportion. After denaturation at 100°C for 10 min, the protein was quickly transferred to ice for cooling. According to the protein concentration, 30–50 μg samples were processed at 60 V for 1 h at a constant pressure and then at 150 V with electrophoresis for 1 h. After PVDF transfer, the membrane was sealed with 5% skim milk for 1–2 h. An appropriate proportion of diluted primary antibody was added, incubated at room temperature for 1 h on a shaker, and then kept overnight at 4°C. The next day, after washing with PBS, horseradish peroxidase-labeled secondary antibody was incubated for 1 h at room temperature. Finally, ECL chemiluminescent solution was added to the PVDF membrane and scanned by a gel imaging system to quantitatively analyze the optical density of the strip.

### FJB Immunofluorescence and TUNEL Staining

Paraffin sections of mouse brain tissue were dewaxed, and FJB working fluid was partially added dropwise overnight at 4°C. Neutral resin was then used to seal the slices. Slices were observed under a Nikon inverted fluorescence microscope, and images were collected. Sections were partially placed in Proteinase K for incubation, washed in PBS, and covered in film. Reagent 1 (TdT) and reagent 2 (dUTP) were added to the TUNEL kit, and the slices were washed again. The two reagents were mixed at a ratio of 1:9. DAPI was used to stain the nucleus, and then the slices were sealed with antifluorescence quenching and imaged under a fluorescence microscope.

### Immunohistochemical Staining

The paraffin section of human brain tissue was treated at 37°C overnight to prevent exfoliation. Paraffin sections were baked at 65°C for half an hour, followed by routine dewaxing and hydration. Slices were immersed in sodium citrate antigen repair solution, boiled for 3 min with a microwave (high fire mode), and cooled at room temperature. The slices were then washed with PBS, soaked in 3% hydrogen peroxide for 20 min at room temperature, and then washed with PBS again. The slices were then covered with immune staining blocking solution at room temperature for 20 min. The NLRP3 antibody (diluted 1:200) was added, and the slices were fixed overnight at 4°C in a wet box. The next day, the slices were rewarmed at 37°C for 45 min and washed in PBS. The slices were then covered with Reagent I (biotin-labeled secondary antibody), incubated at 37°C for 20 min, and washed with PBS five times. Reagent II (horseradish peroxidase-labeled streptomycin egg essence working solution) was then added, and the slices were incubated at 37°C for 20 min and washed with PBS. The tissue was stained with DAB for 2–5 min, the staining degree was observed under the microscope, and they were fully rinsed with tap water. They were then stained with hematoxylin for 2 min, then they were differentiated with hydrochloric acid for 1–2 s, and finally, they were fully rinsed with tap water. The specimen was then dehydrated, cleared, and sealed.

### Detection of IL-1β by ELISA

An IL-1β detection kit was purchased from eBioscience Company (USA). The double antibody sandwich method was used according to the kit instructions. The steps included the following: Samples were added to the enzyme-labeled plate, sealed with film, incubated at room temperature (18–25°C) for 2 h, and then washed five times. Next, 100 μl of enzyme-labeled reagent was added to each well except for the blank wells. The plates were sealed and incubated at room temperature (18–25°C) for 1 h and were then washed five times. Afterward, 100 μl of color developing agent was added to the samples, gently shaken and mixed, and developed color at room temperature without light for 10 min. Finally, 100 μl of the termination solution was added, and the absorbance of each well was measured with the enzyme reader at 450 nm within 10 min of color development; the concentration of IL-1β was calculated according to the standard curve.

## Statistics

Statistical analysis was carried out with the SPSS software (version 16.0). The experimental results were expressed as the mean ± standard deviation. Student's *t*-test was used for comparison of two experimental groups. Single factor regression analysis was used to study the factors related to the severity of status epilepticus. *P* < 0.05 was considered statistically significant.

## Results

### Behavioral Changes

After the intraperitoneal injection of scopolamine, no behavioral abnormalities were observed in the mice. Approximately 10 min after pilocarpine injection, mice exhibited head and neck shaking, and wet dog–like tremors. After ~30 min, mice showed forelimb clonus, rearing, and falling, even including jumps. Seizures were terminated after intraperitoneal injection of 100 g/L of 3 ml/kg chloral hydrate. The surviving mice had paroxysmal head and neck shaking, little food intake, and emaciated bodies.

### Success Rate of Modeling

In the epilepsy group, pilocarpine induced seizures after injection. Twitching stopped on its own in one mouse after 5 min, one mouse died within 3 h, two died within 7 days, and no deaths occurred at 24 h or 3 days. The successful rate of modeling was 87.5% (28/32) after excluding unsuccessful modeling and dead mice.

### Detection of IL-1β

**Figure 1:**
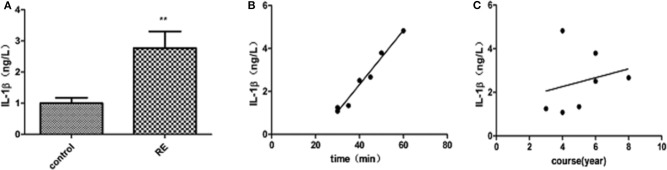
**(A)** ELISA analysis of IL-1β in the peripheral blood of patients with refractory epilepsy and control patients. IL-1β levels in patients with refractory epilepsy increased significantly compared with those in the control group (*t* = 2.813, *P* = 0.01). **(B)** Correlation between peripheral blood IL-1β levels and the duration of a single seizure in patients with refractory epilepsy. IL-1β levels are linearly related to the duration of a single seizure (*r* = 0.9735, *P* < 0.05). **(C)** Correlation between peripheral blood IL-1β levels and the disease course of patients with refractory epilepsy. No correlation was found between IL-1β levels and the disease course of patients. **P < 0.01.

**Figure 2:**
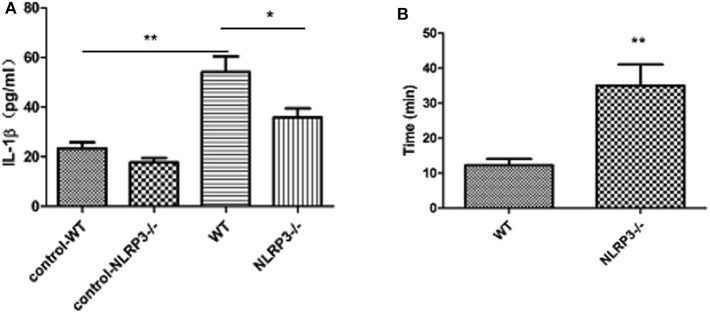
ELISA analysis of IL-1β levels in the peripheral blood of mice in the wild-type group and NLRP3 knockout group 3 days after modeling (*n* = 8). **(A)** The level of IL-1β in the peripheral blood of mice in the wild-type group was significantly higher than that in the control group (*t* = 8.259, *P* < 0.0001) and decreased after NLRP3 gene knockout (*t* = 3.481, *P* = 0.004). **(B)** Comparison of the modeling initiation time of wild-type and NLRP3 knockout mice. The initiation time of NLRP3 gene knockout mice (35 ± 6.075 min) was significantly longer than that of WT mice (12.29 ± 1.796 min, *P < 0.05; **P < 0.01).

IL-1β levels in the peripheral blood of patients with refractory temporal lobe epilepsy were significantly higher than those of the control group (*t* = 2.813, *P* = 0.01). There was also a linear correlation between these levels and the duration of single seizures (*r* = 0.9735, *P* < 0.05) but not with the duration of the disease (see Figure 1). IL-1β levels in the peripheral blood of WT mice were significantly higher than those in the control group 3 days after modeling (*t* = 8.259, *P* < 0.0001). IL-1β levels in the peripheral blood of NLRP3 knockout mice were higher than those of the control group but lower than those of the WT group (*t* = 3.481, *P* = 0.004). The initiation time of NLRP3 knockout mice was 35 ± 6.075 min, which was longer than that of WT mice, 12.29 ± 1.796 min, *P* < 0.05 (see Figure 2).

### Immunohistochemical and Fluorescence Staining

**Figure 3:**
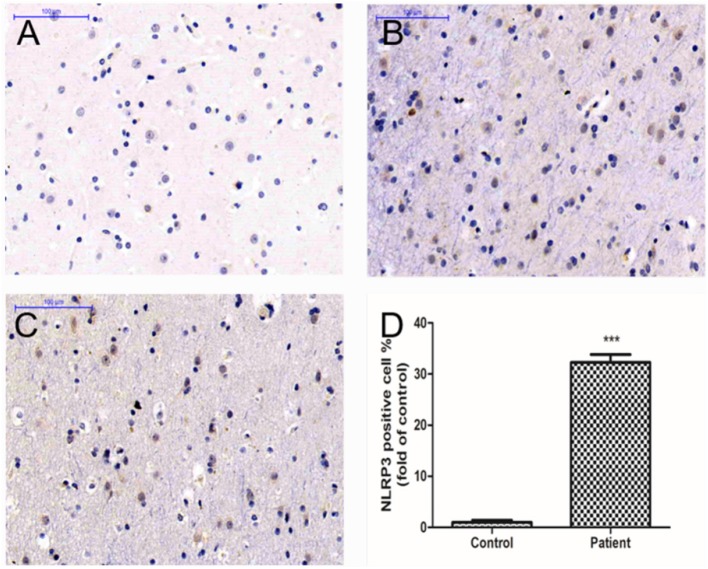
Immunohistochemical staining showed NLRP3-positive cells in the temporal lobe cortical tissues of RE patients. **(A)** Cortical tissues of a control human. **(B,C)** Cortical tissues of RE patients. The brown stained cells are NLRP3-positive cells. **(D)** The mean OD value of NLRP3 was significantly higher in the RE patients than in controls (***P < 0.001).

**Figure 4:**
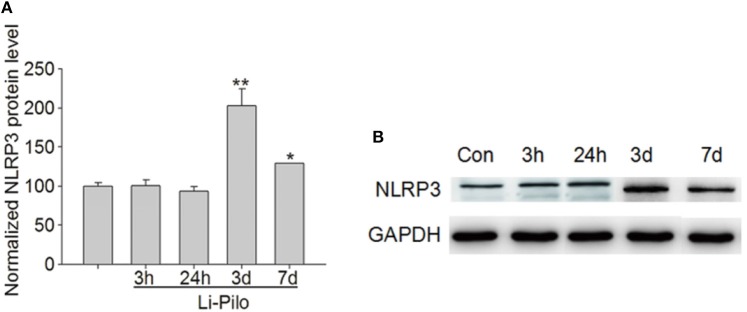
Western blot analysis of NLRP3 expression in the cerebral cortex of wild-type mice. **(A)** Statistical analysis of the mean OD ratios indicated that the content of NLRP3 in the cerebral cortex of wild-type mice did not change significantly at 3 and 24 h after modeling compared with the control group but increased significantly at 3 days and decreased at 7 days to a level that was still higher than that in the control group. **(B)** The expression level of NLRP3 is increased in lithium–pilocarpine-induced epilepsy models (lanes 4–5) compared with that in the control group (lane 1). There was no change in the model group (lanes 2–3). *P < 0.05; **P < 0.01.

**Figure 5:**
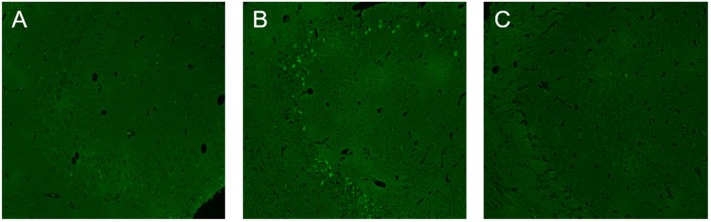
Neuronal necrosis in the CA3 region of the hippocampus in wild-type and NLRP3 knockout mice 7 days after modeling. **(A)** There was little neuronal necrosis within the control group. **(B)** Necrosis in hippocampal neurons increased 7 days after the wild-type mouse model was established, and the green stained cells are necrotic cells. **(C)** Necrosis in hippocampal CA3 neurons decreased significantly after NLRP3 gene knockout.

**Figure 6:**
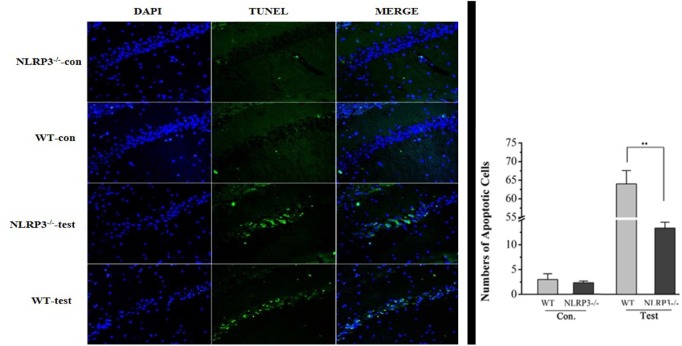
Neuronal apoptosis in the CA3 region of the hippocampus in wild-type and NLRP3 knockout mice 7 days after modeling. Apoptosis in hippocampal neurons increased 7 days after the wild-type mouse model was established, and the green stained cells are apoptotic cells. Apoptosis in hippocampal CA3 neurons improved significantly after NLRP3 gene knockout. **P < 0.01.

The number of NLRP3-positive cells in the temporal lobe of RE patients was significantly higher than that in the control group (see Figure 3). The expression levels of NLRP3 in the cerebral cortex of WT mice did not change significantly at 3 or 24 h after modeling compared with the control group but increased significantly at 3 days and decreased at 7 days, although these levels remained higher than those in the control group (see Figure 4). Necrosis and apoptosis in hippocampal neurons increased 7 days after the WT mouse model was established, and necrosis and apoptosis in hippocampal CA3 neurons improved significantly after NLRP3 gene knockout (see Figures 5, 6).

## Discussion

RE is a condition that is closely related to immune inflammation. It was previously observed that the expression levels of inflammatory factors, such as IL-1β and IL-18 were increased in the serum of epilepsy patients during the interseizure period and that the levels of IL-17A and IFN-γ in the plasma of epileptic patients with different seizure types reflected the severity of epilepsy (16–18). Dubé et al. found that inflammatory cytokines (such as IL-1β) played a crucial role in epilepsy caused by febrile seizures in children (19). Some researchers reported that lipopolysaccharide could alter the epileptic seizure threshold and promote epilepsy in rodents, and this process was involved in the activation of the cytokines IL-1β, tumor necrosis factor α (TNF-α), and COX-2 (20, 21). The IL-1β expression level is low in the brain in normal physiological conditions; however, the IL-1β level increases rapidly when acute brain injury occurs (22). Ravizza et al. investigated that IL-1β/IL-1R1 expression levels were increased in neurons and glial cells of epileptic foci in patients with drug-refractory epilepsy. This phenomenon has also been verified in animal models of epilepsy (23). The findings from Tombini et al. also showed in the group of patients with epilepsy an increase in IL-6 and a decrease in TNF-α with respect to healthy subjects (24).

Additionally, the NLRP3 gene is known to be associated with many non-infectious diseases of the nervous system, such as spinal cord injury and X-linked adrenoleukodystrophy (25, 26). NLRP3 is also involved in the innate immune response of the body (27). These observations suggest that NLRP3 and inflammatory factors are involved in the occurrence and development of epilepsy.

Our study found glial cell proliferation and increased NLRP3 expression levels in the cortex of patients with refractory temporal lobe epilepsy. This finding is consistent with the reported secretion of NLRP3 by microglia (28). Recurrent epileptic seizures resulted in the increased active release of NLRP3 by glial cells. Additionally, the levels of IL-1β in the peripheral blood of patients with intractable epilepsy were higher than those of the control group, which was consistent with the results reported in the literature (29). Serum IL-1β levels were also positively correlated with the duration of a single convulsion but not with the duration of the disease. The results showed that the serum IL-1β levels in the acute stage of convulsion reflected the severity of the convulsion to some extent. A similar relationship can also be detected in animal models. The expression levels of NLRP3 in the cortex and IL-1β in the peripheral blood of mice increased significantly after 3 days of modeling. This finding indicates that repeated epileptic seizures activate NLRP3 inflammatory corpuscles *in vivo*. NLRP3 inflammatory corpuscles promote the activation and release of intracellular proinflammatory factor IL-1β to the extracellular domain, which is involved in the cascade reactions of inflammation.

Some scholars have found that prednisone can inhibit NLRP3 to reduce immune demyelination (30) and that inhibiting NLRP3 can alleviate brain injury from status epilepticus (31). For this reason, we used NLRP3 gene knockout mice to construct a status epilepticus model. The initiation time of the NLRP3 knockout mouse model was significantly longer than that of the WT group, and the level of IL-1β in peripheral blood was lower than that in the WT group. It has been suggested that inhibiting NLRP3 may prolong the latent period of epileptic seizures and reduce the expression levels of IL-1β. Neuron necrosis and apoptosis levels within the hippocampal CA3 region increased significantly 7 days after the WT mouse model was established. After inhibiting NLRP3, neuronal necrosis and apoptosis levels in the same area of the hippocampus were significantly improved. Some scholars have found that curcumin can reduce convulsions by inhibiting NLRP3 to reduce levels of excitatory glutamate in neurons (11). In this study, NLRP3 and IL-1β were found to be involved in the occurrence and development of refractory temporal lobe epilepsy. Inhibiting NLRP3 may play a role in delaying the latent period of convulsion onset and alleviating local brain tissue damage by reducing the expression of inflammatory factors *in vivo*.

Limitations of this study include only investigating the peripheral blood 3 days after modeling and a lack of dynamic observations of the time points of IL-1β elevation. Whether NLRP3 knockout attenuates brain tissue damage in other regions requires further study.

## Data Availability Statement

The raw data supporting the conclusions of this article will be made available by the authors, without undue reservation, to any qualified researcher.

## Ethics Statement

The studies involving human participants were reviewed and approved by Children's Hospital of Nanjing Medical University. The patients/participants provided their written informed consent to participate in this study. The animal study was reviewed and approved by Children's Hospital of Nanjing Medical University.

## Author Contributions

All authors listed have made a substantial, direct and intellectual contribution to the work, and approved it for publication.

### Conflict of Interest

The authors declare that the research was conducted in the absence of any commercial or financial relationships that could be construed as a potential conflict of interest.
